# Transepithelial photorefractive keratectomy - an analysis of the solutions offered by the industry nowadays

**DOI:** 10.22336/rjo.2025.26

**Published:** 2025

**Authors:** Daiana-Andreea Margarit, Mihnea Munteanu, Horia Tudor Stanca

**Affiliations:** 1 Ophthalmology Department, “Victor Babeş” University of Medicine and Pharmacy, Timişoara, Romania; 2 Oftalmo Sensory-Tumor Research Center-ORL (EYEENT), Timişoara, Romania; 3 Ophthalmology Department, “Carol Davila” University of Medicine and Pharmacy, Bucharest, Romania

**Keywords:** TransPRK, SmartPulse, Streamlight, TransEpi PRK, Excimer laser, TransPRK = Transepithelial Photorefractive Keratectomy, PRK = Photorefractive Keratectomy, LASIK = Laser-Assisted in Situ Keratomileusis, SPT = SmartPulse Technology, UDVA = Uncorrected distance visual acuity, CDVA = Corrected distance visual acuity, PTK = Phototherapeutic Keratectomy

## Abstract

**Purpose:**

To review and compare the current commercial solutions for transepithelial photorefractive keratectomy (TransPRK) offered by leading excimer laser manufacturers—SCHWIND eye-tech-solutions (AMARIS with SmartPulse), Alcon (WaveLight EX500), and Bausch + Lomb Technolas (TENEO 317), with a focus on technology, treatment protocols, and clinical outcomes.

**Background:**

TransPRK, a no-touch surface ablation technique, eliminates mechanical or alcohol-based epithelial removal by using the excimer laser to ablate both epithelium and stromal tissue in a single step. This approach offers advantages, including lower epithelial trauma, reduced risk of infection, and faster epithelial healing. Industry innovation has significantly improved TransPRK outcomes through refined epithelial mapping, high-speed ablation, and advanced eye-tracking systems.

All three platforms—SCHWIND AMARIS, WaveLight EX500, and Technolas TENEO—offer effective TransPRK capabilities, each with distinctive advantages. SCHWIND currently leads in terms of surface quality and early visual recovery due to SmartPulse Technology. WaveLight EX500 provides high-speed ablation and robust platform integration, while Technolas TENEO offers customizable epithelial profiles and a user-friendly interface.

**Conclusion:**

In summary, modern TransPRK systems provide safe, effective, and increasingly customizable options for correcting myopia and astigmatism. The choice between platforms depends on a range of factors, including surgical goals, patient profile, available technology, and the surgeon’s familiarity with the system. As laser platforms continue to evolve, further innovations in epithelial mapping, real-time ablation control, and surface smoothing may continue to enhance the outcomes and expand the indications for TransPRK.

## Introduction

Transepithelial Photorefractive Keratectomy is an advanced form of surface ablation in refractive eye surgery. Unlike LASIK, which involves creating a corneal flap, TransPRK uses an excimer laser to remove the corneal epithelium and reshape the underlying stromal surface in a single, touch-free procedure. This technique reduces mechanical stress on the eye, eliminates flap-related risks, and is especially well-suited for individuals with thin corneas, contact lens intolerance, or those with active lifestyles [1].

In response to the growing demand for minimally invasive, high-precision refractive procedures, the ophthalmic industry has developed and refined a variety of laser platforms tailored for TransPRK. Leading technologies include the SCHWIND Amaris system, featuring SmartPulse Technology for smoother ablation and faster visual recovery; Alcon’s WaveLight EX500, which incorporates advanced eye tracking and highly customizable treatment options; and Bausch + Lomb’s Technolas Teneo, offering adaptable treatment modes, including PTK enhancements [2-4].

Recent clinical studies and market feedback indicate that single-step TransPRK procedures—where epithelial removal and stromal ablation are performed simultaneously—offer better patient comfort, faster healing, and fewer postoperative complications compared to traditional two-step PRK or LASIK [1].

The current industry landscape reflects a strong commitment to innovation in TransPRK, with a focus on maximizing visual acuity, improving safety profiles, and broadening patient eligibility. As these technologies continue to evolve, TransPRK is positioned to play a central role in the future of laser vision correction [3].

Refractive surgery has undergone a significant evolution over the past few decades, transitioning from mechanical keratomileusis to exact laser-based procedures. Among these, Transepithelial Photorefractive Keratectomy has emerged as a cutting-edge, no-touch alternative to traditional surface ablation techniques [5]. Unlike traditional PRK, which involves mechanically or chemically removing the corneal epithelium, TransPRK performs both epithelial and stromal ablation simultaneously using excimer laser technology. This innovation streamlines the procedure, enhances patient comfort, accelerates healing, and reduces the risk of postoperative issues, such as pain and corneal haze [6,7].

In response to the increasing demand for safer and more effective refractive procedures, the ophthalmic industry has introduced a range of TransPRK platforms, each distinguished by its specific technology and treatment algorithms. These systems vary in key aspects such as ablation techniques, pulse optimization, eye tracking precision, and the degree of treatment customization—all of which play a significant role in determining clinical outcomes. This paper provides a comparative analysis of the most widely used TransPRK platforms, examining their underlying technologies, benefits, limitations, and their influence on contemporary refractive surgery practices [3].

## History

Transepithelial Photorefractive Keratectomy evolved from the foundational work in surface ablation techniques, particularly Photorefractive Keratectomy (PRK), which Theo Seiler and colleagues pioneered in the late 1980s. As the first form of laser refractive surgery, PRK involved the manual or chemical removal of the corneal epithelium, followed by excimer laser ablation of the stromal layer to correct refractive errors such as myopia and astigmatism [8,9].

Although PRK proved to be clinically effective, it presented several significant drawbacks, including postoperative pain, slower visual recovery, and the potential for corneal haze, primarily stemming from the mechanical or alcohol-assisted removal of the epithelium. These challenges prompted the development of Laser-Assisted in Situ Keratomileusis (LASIK) in the 1990s, which rapidly gained popularity due to its quicker recovery time and reduced patient discomfort [5,9].

However, LASIK introduced its own set of risks, including flap-related complications and a higher likelihood of postoperative dry eye, which renewed interest in refining surface ablation techniques. This led to the development of TransPRK—a “no-touch” evolution of PRK—where the laser itself removes the corneal epithelium, eliminating the need for mechanical instruments or alcohol-based solutions [5].

The first generation of TransPRK procedures used a two-step approach: an initial epithelial ablation was performed using standard laser settings, followed by stromal ablation. Over time, this evolved into a single-step procedure, where both epithelial and stromal tissue are ablated seamlessly using customized ablation profiles. This innovation reduced surgical time, improved refractive accuracy, and significantly enhanced patient comfort and safety [10,11].

By the late 2000s and early 2010s, several excimer laser platforms—such as SCHWIND Amaris, Alcon Wavelight, and Bausch + Lomb’s Technolas Teneo had developed integrated tPRK protocols. These systems incorporated high-frequency tracking, optimized ablation algorithms, and epithelial modeling based on population averages or individual topographies [3,8,10].

Today, TransPRK is widely recognized as a safe, effective, and minimally invasive refractive surgery technique, particularly suited for patients with thin corneas, those with active lifestyles, or those with contraindications to LASIK. The continued refinement of laser technology, ablation strategies, and postoperative care has positioned TransPRK as a highly viable option in modern refractive surgery [10].

### SCHWIND Amaris system

Transepithelial PRK with the SCHWIND Amaris is a single-step, no-touch surface ablation procedure used to correct refractive errors, including myopia, hyperopia, and astigmatism. The SCHWIND Amaris excimer laser removes both the corneal epithelium and stromal tissue in a single laser ablation sequence, without the need for alcohol or mechanical tools. This approach aims to combine the biomechanical safety of PRK with a smoother postoperative experience, improved visual recovery, and a reduced risk of complications [12,13].

### Technological Advancements

The SCHWIND Amaris platform incorporates several key technologies that enhance the efficacy and safety of tPRK:

- SmartPulse Technology (SPT): Improves smoothness of the stromal bed, reduces light scattering and healing irregularities, leads to faster recovery of functional vision postoperatively [1,2].
- SmartSurfACE® Technology: Combines TransPRK with SmartPulse Technology (SPT) to create a smoother ablation surface, promoting faster epithelial healing and better visual quality immediately after surgery, and enhances patient comfort by reducing corneal surface irregularity [1,2].
- Eye Tracking & Ablation Control: 6D eye tracking ensures precise laser delivery even with micro-movements, automatic fluence level adjustment for optimized energy delivery [1,2].

### Clinical Outcomes

Clinical studies have demonstrated the effectiveness of TransPRK using the SCHWIND Amaris system:

- A meta-analysis by Sabau et al. of 16 studies involving 1,924 eyes treated with SCHWIND Amaris TransPRK reported high efficacy (94%), safety (with a 0% complication rate), and predictability (89%) [1,4].
- A study by Tony Ho involving 150 eyes with moderate to high myopia showed that 98% of eyes achieved uncorrected distance visual acuity (UDVA) of 20/25 or better, with no loss of corrected distance visual acuity (CDVA) [1,5].
- A study by Margarit et al. of 92 eyes with mild myopia demonstrated that 96% of eyes achieved postoperative UDVA of 20/20 or better [1,6].

Transepithelial photorefractive keratectomy performed with the SCHWIND Amaris excimer laser system provides a safe, effective, and minimally invasive solution for laser vision correction. Incorporating advanced features like SmartPulse and SmartSurfACE technology, the procedure delivers improved precision and visual outcomes, making it a strong alternative for patients with thin corneas or those unsuitable for LASIK [1,3,16].

### WaveLight EX500

The WaveLight EX500 is a high-speed excimer laser system designed to deliver precise and efficient refractive treatments. It offers advanced features such as eye tracking, pulse control, and customizable ablation profiles, making it suitable for various refractive errors, including myopia, hyperopia, and astigmatism [17,18].

Key technological features include:

- Wavefront-optimized and wavefront-guided treatment modes, allowing tailored correction of refractive errors.
- Fast ablation speed (approximately 1.4 seconds per diopter of correction).
- Multi-dimensional 1050 Hz eye-tracking that follows the eye in real time across five axes.
- Thermal control algorithms that minimize tissue damage during treatment.
- High-frequency pulse delivery, ensuring smooth ablation and reduced thermal load [18].

A notable clinical study by Zhang et al. (2021) titled “Two-Step Transepithelial Photorefractive Keratectomy with WaveLight EX500 Platform for Adolescents and Adults” evaluated the safety and effectiveness of EX500-based TransPRK in correcting low to moderate myopia. Key findings include:

- Visual Outcomes: At 12 months postoperatively, nearly 49% of treated eyes achieved a spherical equivalent within ± 0.50 D of the target refraction. Most eyes achieved UDVA of 20/25 or better.
- Stability and Safety: The procedure was found to be safe and stable, with no significant regression or late-onset complications.
- Complications: Transient haze (grade 0.5-1.0) was reported in a minority of patients but resolved by the one-year follow-up in most cases [17].

Transepithelial PRK, using the WaveLight EX500 excimer laser (Streamlight™), represents a significant advancement in surface ablation techniques. It offers an elegant, safe, and effective alternative to LASIK, particularly for patients with thinner corneas or a higher risk of dry eye. With high-speed ablation, advanced eye tracking, and reliable visual outcomes, the EX500 is a powerful tool in modern refractive surgery [17,18].

#### Technolas Teneo 317 Model 2

The Technolas Teneo 317 Model 2 is a compact and advanced excimer laser system designed for refractive surgeries, including TransPRK [4]. Key features include:

- High-Speed Performance: Operates at 1,740 Hz with a treatment time of approximately 1 second per diopter in PROSCAN ECO mode.
- Advanced Eye Tracking: Utilizes a 3-axis, 360° swiveling microscope and a high-speed eye tracker to ensure precise alignment and treatment delivery.
- Optimized Workflow: The transEpi PRK procedure combines phototherapeutic keratectomy (PTK) for epithelial removal and photorefractive keratectomy (PRK) for refractive correction into a single, efficient step [4].

A clinical study by Niknam et al. titled “Epithelial Thickness Map-Adjusted Transepithelial Photorefractive Keratectomy for Treatment of Myopic Astigmatism: 12-Month Results” evaluated the refractive results of tPRK using the Technolas Teneo2 Excimer laser platform (TransEpi PRK). The study included 199 patients with myopia ranging from -1 to -7 diopters [19]. Key findings at the 12-month follow-up include:

- Uncorrected Distance Visual Acuity: 20/20 in all patients.
- Spherical Equivalent Refraction: The 12-month post-operative spherical equivalent refraction was 0.90 ± 0.33 D, 0.79 ± 0.26 D, and 0.60 ± 0.19 D in groups targeting 0 D, -0.25 D, and -0.5 D, respectively [19] (**Table 1**).

**Table 1:** Comparative Table: Industry Solutions for Transepithelial PRK

Feature	SCHWIND Amaris	WaveLight EX500 (Alcon)	Technolas Teneo (B+L)
**Ablation Speed**	500-1050 Hz (depending on model)	500 Hz	Up to 1740 Hz (Model 2)
**Eye Tracking**	6D high-speed eye tracking	1050 Hz multidimensional tracking	3D high-speed eye tracker with 360° microscope
**Epithelial Mapping**	Based on population averages or optional custom maps	Nomogram or topography-based (depending on software)	Optional epithelial thickness map integration
**Key Technologies**	SmartSurfACE, SmartPulse Technology	Wavefront-optimized, wavefront-guided, Custom-Q	transEpi PRK, PROSCAN ECO mode
**tPRK Mode**	True single-step (no-touch) SmartSurfACE	Typically, two-step, but can be streamlined	Two-step with PTK + PRK or integrated transEpi mode
**Healing Time**	2-3 days (faster with SmartPulse)	3-4 days	3-5 days
**Postop Pain**	Mild (reduced by SmartPulse smooth ablation)	Mild to moderate	Mild to moderate
**Clinical Outcomes (UDVA ≥ 20/20)**	~94-98% depending on the degree of myopia	~90-95% for low to moderate myopia	~95-100% reported in some studies
**Advantages**	Very smooth ablation, fast healing, ideal for irregular corneas	Fast, reliable, and well-integrated into many clinics	High-speed platform, compact design, intuitive GUI
**Limitations**	High cost, requires dedicated software integration	Slight undercorrection tendency in high myopia	Less global clinical literature compared to others

## Discussion

The evolution of transepithelial photorefractive keratectomy highlights the ophthalmic industry’s commitment to advancing safer, more efficient, and less invasive vision correction techniques. Leading this progression are three prominent excimer laser platforms: the SCHWIND Amaris, Alcon WaveLight EX500, and Bausch + Lomb’s Technolas Teneo - each offering a unique yet practical approach to tPRK. While all three systems utilize no-touch, all-laser methods to simplify the procedure, their clinical performance and advantages differ due to variations in engineering design, software capabilities, and ablation strategies [2-4].

The SCHWIND Amaris platform is often considered the benchmark for surface ablation refinement, particularly due to its SmartPulse and SmartSurfACE technologies. These features significantly improve stromal bed smoothness and reduce epithelial healing time. Clinical outcomes consistently demonstrate rapid recovery and excellent visual acuity, even in challenging cases involving thin or irregular corneas. The system’s true single-step ablation of both the epithelium and stroma delivers a fully no-touch procedure with minimal thermal impact, potentially reducing postoperative discomfort and accelerating visual rehabilitation. However, incorporating this technology into routine surgical practice may present challenges, including a steeper learning curve and significant upfront costs, which could limit adoption in smaller or general ophthalmic practices [2,13-1,6].

In contrast, the WaveLight EX500, though not initially built for tPRK, has proven to be a highly adaptable and dependable platform. It is widely used due to its wavefront-optimized treatments, fast ablation speed, and high-frequency multidimensional eye tracking. While TransPRK procedures on the EX500 are commonly performed in two steps (PTK + PRK), many centers have implemented workflow optimizations to simulate a near-seamless epithelial removal and stromal correction process. Studies show strong outcomes in terms of safety and efficacy, though there is a slight trend toward mild undercorrection, especially in higher myopic corrections. Nonetheless, its widespread adoption and robust software support make it an appealing option for high-throughput refractive practices [3,17,18].

The Technolas Teneo 317 Model 2 from Bausch + Lomb offers the fastest ablation rates among the three systems and is specifically designed to accommodate efficient transEpi PRK workflows. Its ability to integrate epithelial mapping and high-speed ablation, combined with a user-friendly interface, makes it particularly suitable for busy surgical environments. Clinical data reveal excellent visual outcomes and low complication rates, even in moderate to high myopia. However, the literature supporting the Teneo platform in TransPRK remains comparatively limited, and broader clinical adoption is still under development in many regions. Nevertheless, its promising results and speed-focused design suggest a strong potential for growth in the tPRK space [3,4,19].

## Conclusion

Overall, each system demonstrates a high level of safety, efficacy, and predictability in TransPRK applications. The choice between platforms often depends more on institutional goals and surgeon preference than on significant performance differences. The SCHWIND Amaris may be ideal for practices prioritizing precision and epithelial preservation; the WaveLight EX500 for versatility and proven reliability; and the Technolas Teneo for speed and operational efficiency. Future comparative studies—particularly randomized clinical trials—will be essential to further clarify their respective strengths in diverse clinical settings.
